# Biochemical and Functional Changes in the Eye As a Manifestation of Systemic Degeneration of the Nervous System in Parkinsonism

**Published:** 2018

**Authors:** A. R. Kim, T. A. Pavlenko, L. A. Katargina, N. B. Chesnokova, M. V. Ugrumov

**Affiliations:** Koltzov Institute of Developmental Biology of Russian Academy of Sciences, Vavilov Str., 26, Moscow, 119334, Russia; Helmholtz Moscow Research Institute of Eye Diseases of Ministry of Health of the Russian Federation, Sadovaya-Chernogryazskaya Str., 14/19, Moscow, 105062, Russia; National Research University Higher School of Economics, Myasnitskaya Str., 20, Moscow, 101000 , Russia

**Keywords:** Parkinson’s disease, neurodegeneration, non-motor symptoms, experimental models

## Abstract

Parkinson’s disease (PD) is a systemic neurodegenerative condition caused by the death of dopaminergic neurons of the nigrostriatal system of the brain. This disease is diagnosed after most neurons have already been lost, which explains the low efficiency of treatment. Hope for increasing treatment efficiency rests in the development of new strategies for early diagnosis of PD based on a search for peripheral markers that appear as early changes in non-motor functions. Since impairment of the visual function is one of the manifestations of PD, the purpose of our work was to identify biochemical and physiological changes in a mouse’s eye and eyelid in models of preclinical (presymptomatic) and clinical (symptomatic) stages of PD. We found that the norepinephrine, dopamine, and serotonin levels in the mouse eye reduced not only in the model of the early clinical stage, but also in the model of preclinical stage, an indication that pathological changes in the monoaminergic systems of the brain had affected the eye even before the motor disorders emerged. Moreover, in both models of PD, mice had increased intraocular pressure, indicating the development of both metabolic and functional impairments, which can be used as diagnostic markers. Unlike in the eye, the serotonin level in the eyelid was increased in mice at both parkinsonism stages and in presymptomatic mice to a much higher extent than in symptomatic ones. Given that serotonin is involved in the regulation of lacrimal glands of the eyelid, an increase in its level in parkinsonian mice should alter the composition of tear fluid, which could serve as a diagnostic marker of early stage of PD. Thus, the changes in the metabolism of monoamines in the eye and eyelid observed in mice at the early stage of parkinsonism are accompanied by changes in the function of these structures and, therefore, can be used as diagnostic markers of the early stage of PD.

## INTRODUCTION

Parkinson’s disease (PD) is a widespread neurodegenerative disorder caused by the degeneration of the nigrostriatal system of the brain, the key element in the regulation of the motor function. Today, PD is diagnosed based on the presence of motor symptoms in the form of tremor or akinetic rigid syndrome, which appear only many years after the onset of the pathological process, when most nigrostriatal dopaminergic neurons have already been lost. The high degree of degradation of the nigrostriatal system and exhaustion of the compensatory reserves of the brain by the time of the diagnosis are the reason why conventional substitution therapy with dopamine agonists proves to be ineffective [1]. Therefore, there is a pressing need for developing early (preclinical) diagnosis of PD long before the appearance of motor symptoms, as this could make it possible to use neuroprotective therapy to slow down or even stop neurodegeneration [1].

The existing approach to the development of early diagnosis of PD is based on the idea that the disease is systemic; its non-motor symptoms caused by the impaired function of both brain regions beyond the nigrostriatal system and the peripheral nervous system appear long before the motor disorders [1, 2]. It is assumed that complex preclinical diagnosis of PD can be developed on the basis of early changes in non-motor functions and the corresponding changes in body fluids (cerebrospinal fluid and blood) [1].

Impairment of visual analyzer functions and auxiliary eye structures is one of the systemic manifestations of PD. For instance, at the clinical stage patients develop hallucinations, impaired eye and eyelid movement, and reduced amount and altered composition of tear fluid [3-5]. In addition, the dry eye symptom, blepharitis (bilateral recurrent inflammation of the part of the eyelid where the eyelashes grow), changes in eye accommodation (pupillary response to light), impaired secretion and outflow of intraocular fluid, reduced visual acuity, scotoma formation (areas in the field of vision where vision is either completely degenerated or partially diminished), and thinning of the retina layers, in particular, due to the reduction in the number of nerve fibers, are often observed in PD patients [5-7].

Impairments of neurotransmission and the metabolism of monoamines (primarily catecholamines) play a significant role in pathological changes in the visual system in PD, as they alter the monoamine content in eye tissues and intraocular fluid [8-12]. These changes are quite typical, since monoamines are involved in the transmission of visual information in the retina and affect the retinal and choroidal vascular tone [13-15]. In addition, catecholamines in the anterior part of the eye regulate the accommodation rate [16, 17] and the level of intraocular pressure (IOP) by controlling the secretion and outflow of intraocular fluid [18, 19]. In addition to the eyes, eyelids also undergo changes in PD, since they contain numerous glands (glands of Krause and Wolfring, meibomian glands, etc.), with their secretory product being a component of the fluid. The conjunctiva covering the inner surface of eyelids and the glands within it are sympathetically innervated [20, 21]. This innervation is impaired in PD [2], which may be the cause behind blepharitis and the changes in the composition of tear fluid.

It can be assumed that at least some of the abovementioned changes in the eye and eyelids diagnosed at the clinical stage of PD, i.e. after the onset of motor function disorders, are typical of patients at the preclinical stage, prior to motor impairments. This would allow to use these changes as diagnostic markers of the preclinical stage of the disease. This assumption can be verified only by using an experimental model of PD, since it is not yet possible to identify preclinical stage patients. Moreover, both the models of preclinical and clinical stages of PD need to be used to make sure that the model correctly reproduces at least the biochemical and physiological changes in the eye and eyelids observed in patients.

The aim of the current study is to identify early biochemical and physiological changes in the eye in the experimental model of PD. To achieve this, we measured IOP and evaluated the content of monoamines and metabolites in the eye and eyelid tissues in a neurotoxic mice model of the preclinical and early clinical stages of PD.

## EXPERIMENTAL

A total of 98 male C57BL/6 mice aged 2–2.5 months (weight, 22–26 g) purchased from the Pushchino animal facility were used in the study. The animals were kept under standard conditions (22 ± 1°C, light from 8.00 a.m. to 8.00 p.m.) with free access to food and water. The model of the preclinical stage of PD was reproduced by two subcutaneous injections of 1-methyl-4-phenyl-1,2,3,6-tetrahydropyridine (MPTP) (Sigma, USA) at a single dose of 8 mg/kg. The model of early clinical stage was reproduced by four subcutaneous injections of MPTP at a single dose of 10 mg/ kg. The interval between the injections was 2 h in both cases [22]. Animals in the control groups were given injections of 0.9% NaCl using the same scheme.

Prior to MPTP administration, the mice were assessed using a PhenoMaster animal behavior analysis system (TSE Systems, Germany) according to the total distance they traveled during the open-field test. The motor behavior of the animals was evaluated again two weeks after administration of MPTP or NaCl.

Two weeks after MPTP administration, IOP was measured 3 times in the animals using an automatic Tonovet veterinary tonometer (Icare, Finland) and the mean value was calculated. After the IOP measurements, material for a biochemical analysis was collected: the animals were decapitated, and the upper and lower eyelids were isolated. Eye specimens were then prepared by removing the lens and the vitreous body. The eye and eyelid specimens were weighed, frozen in liquid nitrogen, and stored at -70°C for further biochemical analysis.

The dorsal striatum was isolated from the mouse brain according to the previously described procedure used to assess the degree of dopamine decline in the nigrostriatal system of experimental animals [22]. Striatum samples were weighed, frozen in liquid nitrogen, and stored at -70°C for further biochemical analysis.

The content of biogenic amines and their metabolites (norepinephrine, dopamine, serotonin, *L*-dihydroxyphenylalanine (*L*-DOPA), and 5-hydroxytryptophan (5-HTP)) was measured using high-performance liquid chromatography with electrochemical detection (HPLC-ED). The samples were homogenized using a Labsonic M ultrasonic homogenizer (Sartorius, France) in 200 μl of 0.1 N HClO_4_ (Sigma, USA) containing 3,4-dihydroxybenzylamine (DHBA, Sigma) as an internal standard at a concentration of 25 pmol/ml and then centrifuged at 2,000 g for 20 min.

HPLC separation was performed on a reversed-phase ReproSil-Pur column, ODS-3, 4 × 100 mm with pore diameter of 3 μm (Dr. Majsch GMBH, Germany) at a temperature of 30°C and a flow rate of 1.2 ml/min maintained by an LC-20ADsp liquid chromatograph (Shimadzu, Japan). The mobile phase consisted of 0.1 M citrate-phosphate buffer, 0.3 mM sodium octanesulfonate, 0.1 mM EDTA, and 9% acetonitrile (all reagents purchased from Sigma); pH 2.5. Decade II electrochemical detector (Antec Leyden, Netherlands) was equipped with a glassy carbon working electrode (+0.85 V) and an Ag/AgCl reference electrode. The peaks of biogenic amines and metabolites were identified according to their retention times in the standard solution. The content of the substances was evaluated by the internal standard method using the ratio between the peak areas in the standard solution and in the test sample using the LabSolutions software (Shimadzu, Japan).

Statistical analysis of the results was carried out using Student’s t-test and the Statistica software package. *p *≤ 0.05 was considered to be statistically significant.

## RESULTS

Dopamine concentration in the mice striatum was decreased compared to the control values: down to 43.3% in the preclinical PD model and 20.1% in the model of the early clinical stage of PD (*Table*).

Meanwhile, the analysis of motor activity showed no differences in the total distance in the open-field test prior to MPTP or NaCl administration between the control and both study groups (*Table*). However, a 48.2% decrease in the total distance was observed in the model of the early clinical stage of PD compared to the control after MPTP administration (*Table*).

**Fig. 1:**
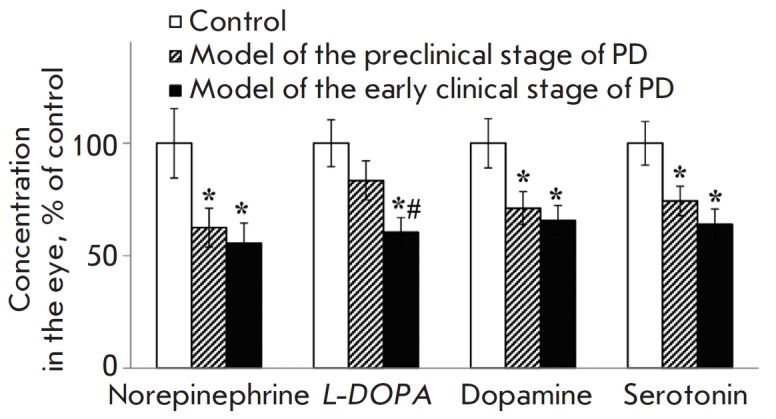
Norepinephrine, *L*-DOPA, dopamine and serotonin concentrations in the mouse eye in the preclinical and clinical models of PD. * *p *≤ 0.05 in comparison to the control mice; # *p *≤ 0.05 in comparison to the preclinical model

**Fig. 2:**
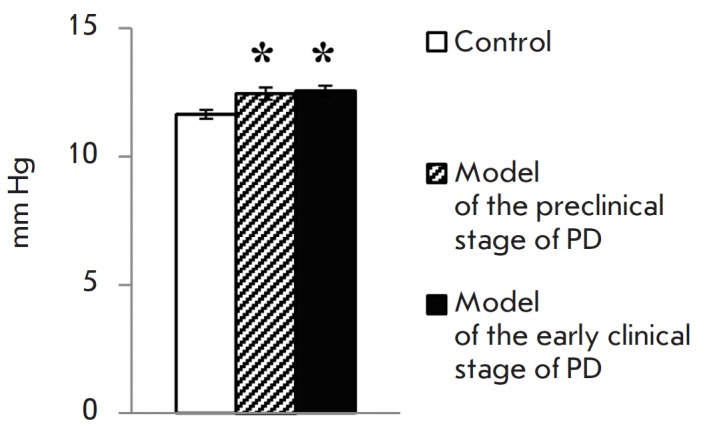
Inctraocular pressure in the preclinical and clinical mouse models of PD. * *p *≤ 0.05 in comparison to the control mice

When modeling the preclinical stage of PD, the norepinephrine, dopamine, and serotonin levels in the eye decreased by an average of 37%, 29% and 26%, respectively, compared to the control. Furthermore, the *L*-DOPA level tended to go down (*p *≤ 0.12) (*Fig. 1*). These changes were even more pronounced in the model of the early clinical stage of PD: the norepinephrine, dopamine, serotonin, and *L*-DOPA levels in the eye were reduced by 44%, 34%, 36%, and 40%, respectively, as compared to the control. Meanwhile, *L*-DOPA concentration in the eye was significantly lower in the mouse model of the early clinical stage of PD compared to that in the preclinical stage model (*Fig. 1*). In addition to the described biochemical changes, a small but statistically significant increase in IOP was revealed in mouse models of both stages of PD (*Fig. 2*).

**Table:** Dopamine level in the striatum and the total distance in open-field test in the models of preclinical and early clinical stages of PD

Group		Dopamine level in the striatum	Total distance (open-field test)	
			prior to administration of MPTP/NaCl	after administration of MPTP/NaCl
		% of the control value		
Control (0.9% NaCl)		100 ± 2.0	100 ± 7.0	95.4 ± 8.8
PD model	at the preclinical stage (2×8 mg/kg MPTP)	43.3 ± 2.4^*^	101 ± 5.6	92.3 ± 6.8
	at the early clinical stage (4×10 mg/kg MPTP)	20.1 ± 2.5^*^	98 ± 6.1	51.8 ± 6.3^*^

^*^p ≤ 0.05 compared to the control.

**Fig. 3:**
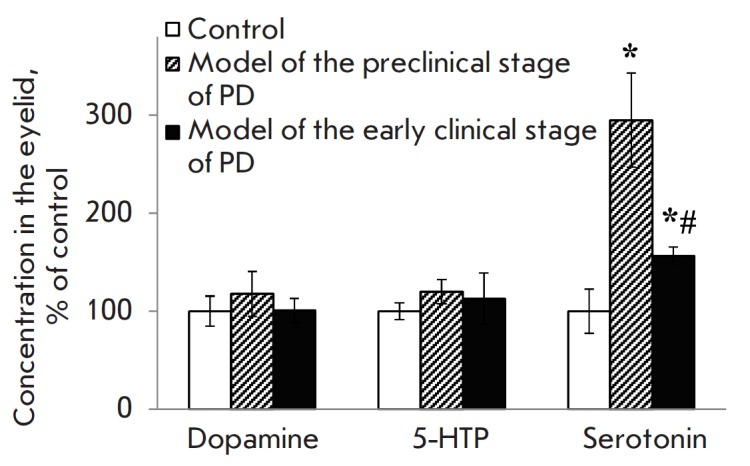
Dopamine, 5-HTP and serotonin concentrations in the mouse eyelid in the preclinical and clinical models of PD. * *p *≤ 0.05 in comparison to the control mice; # *p *≤ 0.05 in comparison to the preclinical model

An increased serotonin level was observed in mice eyelids in the models of both preclinical and early clinical stages of PD, with the level varying among the stages. A 3-fold increase in the serotonin level was observed at the preclinical stage, while an increase of approximately 56% was noted at the clinical stage (*Fig. 3*). However, the levels of dopamine and the precursor of serotonin, 5-HTP, in eyelids in both models of PD stages did not differ from the control values (*Fig. 3*).

## DISCUSSION

**Characterization of the experimental model of Parkinson’s desease**

An important feature of PD is that it has an estimated threshold of neurodegeneration at which motor symptoms occur, meaning the disease proceeds from the preclinical to the clinical stage. This threshold is equal to the death of 50–60% of the cell bodies of dopaminergic nigrostriatal neurons, 70–80% of their axons in the striatum, and loss of 70–80% of dopamine in the striatum in comparison to the control [1]. Since the death of axons of dopaminergic neurons in the striatum precedes the loss of the cell bodies of these neurons in the substantial nigra [1], the following key character istics of the experimental model of PD can be distinguished at different stages:

1. The preclinical stage is characterized by the absence of changes in the total distance in the open-field test, and a less than 70% decrease in the dopamine level in the striatum.

2. The early clinical stage is characterized by a decrease in the total distance in the open-field test and a more than 80% decrease in the dopamine level in the striatum.

We previously demonstrated a 56% reduction in the dopamine level in the striatum in a model of advanced preclinical stage of PD using two injections of MPTP at a single dose of 12 mg/kg; in the early clinical stage model of PD (four injections of MPTP at the same single dose), the dopamine level decreased by 75% compared with the control [22]. The MPTP doses used in this study for PD modeling in mice purchased from the Pushchino animal facility are slightly different from those we described earlier for animals from the Stolbovaya facility [22]. It is known that mice of the same line but obtained from different facilities may differ in sensitivity to MPTP [23]. Therefore, we additionally evaluated the aforementioned key characteristics of the model used in the study to make sure that it fully complied with the model described previously.

In the models of preclinical and clinical stages of PD, the dopamine level in the murine striatum was decreased by 57% and 80%, respectively (*Table*), in comparison to the control group; this finding almost completely coincides with the previously obtained data [22]. A 48% reduction in the total distance in the open-field test as compared to the control (*Table*) was found for the model of clinical stage of PD, which is similar to the motor dysfunctions observed in the previous study [22]. In addition, changes in motor activity for the animal model of preclinical stage of PD were noted neither in the present nor in the previous study (*Table*) [22].

Thus, the doses and schemes for MPTP administration used in this study completely reproduce the previously described mouse models of preclinical and early clinical stages of PD.

**Changes in the metabolism of monoamines in eye tissues and physiological manifestations**

The reduced content of monoamines (norepinephrine, dopamine and serotonin) detected in mouse eyes in the PD models of both early clinical and preclinical stages indicates that the systemic pathological processes developing in PD [2] spread to the eye and appear already at an early stage of PD, before motor symptoms emerge. These results are in good agreement with the published data stating that the dopamine level decreases in the retina of mice with MPTP-induced parkinsonism [24]. It is interesting that no changes in the plasma level of norepinephrine as compared to the control were detected in the mouse models of both PD stages, unlike the situation with the norepinephrine level in the eyes [25]. This indicates that the decrease in the norepinephrine level in the eye in the PD model is region-specific.

Unlike the monoamine level, the *L*-DOPA concentration was reduced only at the clinical stage of PD. The absence of changes in the *L*-DOPA concentration at the preclinical stage of PD indicates that early pathological changes in the eye do not affect the metabolism of the key precursor of catecholamines.

Special attention should be given to the correlation between the decrease in dopamine levels in the striatum as a result of degradation of dopaminergic axons and in mouse eyes at the preclinical and clinical stages of PD, which indirectly confirms the systemic nature of PD pathogenesis. Dopamine level in these animals decreases not only in the striatum, but also in the substantia nigra, the area where the cell bodies of dopaminergic neurons are localized. However, neuronal degeneration in this brain area occurs in mice only at the clinical stage of PD [1, 22].

From a physiological point of view, it is of great interest that both stages of PD in mice are characterized by a slight but statistically significant increase in IOP (*p *≤ 0.015), indicating that functional disorders in the eye emerge prior to the occurrence of motor dysfunctions. It is quite likely that an increase in IOP may result from a reduced catecholamine level in eye tissues. Indeed, the IOP value depends on the rate of intraocular fluid secretion and outflow. Dopamine is involved in the regulation of these processes, as it interacts with the DA2 and DA3 receptors expressed in postganglionic sympathetic nerve endings and reduces the secretion of intraocular fluid, while the interaction between dopamine and the DA1 receptors localized in the ciliary body increases the secretion of intraocular fluid [19]. It is very likely that elevated intraocular pressure could be one of the earliest signs of PD in humans, and it’s rather tempting to use this as a marker for early diagnosis. This possibility is indirectly confirmed by the increased incidence of glaucoma with elevated intraocular pressure in patients with PD compared to the age control [7].

It is yet impossible to understand whether the increase in IOP found in mouse models of PD is characteristic of patients, since this indicator in patients was assessed only at the advanced clinical stage after prolonged treatment with dopamine agonists [26]. In fact, in contrast to mice, the IOP level was reduced in patients. The final answer to the question of whether and how IOP is altered in PD patients can be obtained only after assessing this parameter in treatment-naïve patients at the early clinical stage of PD.

**Changes in the metabolism of monoamines in eyelid tissues**

The significant increase in serotonin level observed in the eyelid confirms its reportedly important role in the development of neuroinflammation in PD [27, 28]. There are two possible explanations for this increase. First, the synthesis of serotonin (mast cells being its source in the eyelid) [29] can increase under the influence of substance P, a neuroinflammation factor whose content rises in the central nervous system in PD [30, 31]. Second, the elevated serotonin level in the eyelid can be rooted in its impaired metabolism; for example, resulting from a reduced activity of N-acetyltransferase, the rate-limiting enzyme for the conversion of serotonin to melatonin. Indeed, a decrease in the activity of this enzyme is considered to be a risk factor for the development of PD [32].

Serotonin is involved in the regulation of the microcirculation of tear fluid and secretory activity of the lacrimal glands localized in the eye conjunctiva [33-36]. Therefore, a significant increase in the serotonin level in mouse eyelids can alter tear composition and cause pathological disturbances in its microcirculation [36], which can lead to the development of the dry eye symptom characteristic of PD [37, 38]. Hence, special attention should be given to an evaluation of tear fluid composition for the development of a preclinical diagnosis of PD. So far, only data on the increase in the TNF-α level in the tear fluid of PD patients are available [39]. Taking into account the important role played by serotonin in the regulation of tear fluid composition on the one hand and the significant difference in the serotonin level in mice eyelid between the models of preclinical and clinical stage of PD on the other hand, one can expect a significant variation in tear fluid composition in patients at preclinical and clinical stages. If this assumption is confirmed after the analysis of the tear fluid of mice in models of preclinical and clinical stages of PD, the changes in tear fluid composition at the preclinical stage could be considered as potential diagnostic markers of the early stage of PD.

Thus, neurotoxic modeling of the preclinical and clinical stages of PD in mice has revealed changes in the monoamine metabolism in the eye and eyelid. These changes are accompanied by an alteration of the functions of these structures and can be used as diagnostic markers of early PD, before the appearance of motor symptoms.

## Abbreviations

5-HTP: 5-hydroxytryptophan
DHBA: 3,4-dihydroxybenzylamine
HPLC-ED: high-performance liquid chromatography with electrochemical detection
IOP: intraocular pressure
L-DOPA: L-3,4-dihydroxyphenylalanine
MPTP: 1-methyl-4-phenyl-1,2,3,6-tetrahydropyridine
PD: Parkinson’s disease
